# Supplementary material for: Low coverage of HIV testing among adolescents and young adults in Nigeria: Implication for achieving the UNAIDS first 95
Source: PLoS One. 2020 May 19;15(5):e0233368. doi: 10.1371/journal.pone.0233368 (PMC7237011; doi:10.1371/journal.pone.0233368)
Supplement: S1 Table — (DOCX) [file pone.0233368.s001.docx]

**Table: S 1** HIV Testing by States

| States | Ever tested for HIV (%) | Recently tested for HIV (%) | HIV prevalence rate (%) |
| --- | --- | --- | --- |
| FCT Abuja | 47.3 | 28.8 | 1.6 |
| Abia | 19.6 | 10.3 | 2.1 |
| Adamawa | 25.8 | 19.5 | 1.2 |
| Akwa Ibom | 41.3 | 25.5 | 5.5 |
| Anambra | 29.3 | 11.4 | 2.4 |
| Bauchi | 8.7 | 2.9 | 0.5 |
| Bayelsa | 31.8 | 13.8 | 1.9 |
| Benue | 61.2 | 49.7 | 5.3 |
| Borno | 17.3 | 3.4 | 1.2 |
| Cross River | 38.8 | 27.1 | 2 |
| Delta | 23.4 | 10.9 | 1.9 |
| Ebonyi | 26.8 | 8.4 | 0.8 |
| Edo | 30.2 | 14.0 | 1.9 |
| Ekiti | 28.6 | 10.5 | 0.8 |
| Enugu | 34.6 | 13.6 | 2 |
| Gombe | 22.8 | 11.3 | 1.3 |
| Imo | 34.4 | 17.5 | 1.8 |
| Jigawa | 2.1 | 0.4 | 0.3 |
| Kaduna | 32.3 | 14.4 | 1.1 |
| Kano | 10.5 | 4.4 | 0.6 |
| Katsina | 6.1 | 1.5 | 0.3 |
| Kebbi | 9.0 | 5.0 | 0.6 |
| Kogi | 23.5 | 10.1 | 0.9 |
| Kwara | 25.9 | 11.6 | 1 |
| Lagos | 39.1 | 22.1 | 1.4 |
| Nasarawa | 47.0 | 29.1 | 2 |
| Niger | 13.7 | 3.6 | 0.7 |
| Ogun | 18.8 | 7.3 | 1.6 |
| Ondo | 14.9 | 7.4 | 1.1 |
| Osun | 20.0 | 11.2 | 0.9 |
| Oyo | 23.1 | 9.9 | 0.9 |
| Plateau | 30.2 | 14.0 | 1.6 |
| Rivers | 44.8 | 32.1 | 3.8 |
| Sokoto | 9.1 | 5.1 | 0.4 |
| Taraba | 27.6 | 15.5 | 2.9 |
| Yobe | 11.9 | 6.5 | 0.4 |
| Zamfara | 7.8 | 4.1 | 0.5 |
